# A Web-Based Rendering Application for Communicating Dental Conditions

**DOI:** 10.3390/healthcare9080960

**Published:** 2021-07-29

**Authors:** Hudson D. Spangler, Miguel A. Simancas-Pallares, Jeannie Ginnis, Andrea G. Ferreira Zandoná, Jeff Roach, Kimon Divaris

**Affiliations:** 1 Division of Pediatric and Public Health, Adams School of Dentistry, University of North Carolina at Chapel Hill, Chapel Hill, NC 27599-7450, USA; simancas@email.unc.edu (M.A.S.-P.); jeannie_ginnis@unc.edu (J.G.); Kimon_Divaris@unc.edu (K.D.); 2 Department of Comprehensive Care, School of Dental Medicine, Tufts University, Boston, MA 02111, USA; andrea.zandona@tufts.edu; 3 Department of Research Computing, University of North Carolina at Chapel Hill, Chapel Hill, NC 27599-7032, USA; jeff_roach@unc.edu; 4 Department of Epidemiology, Gillings School of Global Public Health, University of North Carolina at Chapel Hill, Chapel Hill, NC 27599-7400, USA

**Keywords:** dentistry, communication, digital, 3D, pediatric dentistry

## Abstract

The importance of visual aids in communicating clinical examination findings or proposed treatments in dentistry cannot be overstated. Similarly, communicating dental research results with tooth surface-level precision is impractical without visual representations. Here, we present the development, deployment, and two real-life applications of a web-based data visualization informatics pipeline that converts tooth surface-level information to colorized, three-dimensional renderings. The core of the informatics pipeline focuses on texture (UV) mapping of a pre-existing model of the human primary dentition. The 88 individually segmented tooth surfaces receive independent inputs that are represented in colors and textures according to customizable user specifications. The web implementation SculptorHD, deployed on the Google Cloud Platform, can accommodate manually entered or spreadsheet-formatted tooth surface data and allows the customization of color palettes and thresholds, as well as surface textures (e.g., condition-free, caries lesions, stainless steel, or ceramic crowns). Its current implementation enabled the visualization and interpretation of clinical early childhood caries (ECC) subtypes using latent class analysis-derived caries experience summary data. As a demonstration of its potential clinical utility, the tool was also used to simulate the restorative treatment presentation of a severe ECC case, including the use of stainless steel and ceramic crowns. We expect that this publicly available web-based tool can aid clinicians and investigators deliver precise, visual presentations of dental conditions and proposed treatments. The creation of rapidly adjustable lifelike dental models, integrated to existing electronic health records and responsive to new clinical findings or planned for future work, is likely to boost two-way communication between clinicians and their patients.

## 1. Introduction

Improved communication has significant potential to improve healthcare; arguably, better informed patients are more likely to be engaged in the management of their health conditions, make more informed and better decisions, and ultimately contribute to higher quality of care [1]. A recent systematic review and meta-analysis of the effects of using visual aids to convey health information on patient and consumer health behaviors and outcomes found that the use of pictures improves knowledge, understanding, and recall [2]. The efficacy of visual aid-based interventions aiming to improve patient comprehension and education in the context of surgical procedures and chronic disease management has been demonstrated in numerous clinical trials [3,4]. Conventional visual aids such as pictographs [5], video clips [6,7], and physical models [8,9] have long-recognized merits and are useful adjuncts to written information, especially for low literacy individuals and for contexts where visualization is of the essence. Building upon these modalities, new technologies offer additional opportunities for enhanced means of visual communication. Web-based [10] and multimedia-augmented [11] patient education tools have been tested in randomized controlled trials in clinical and surgical settings with positive results. Such communication aids are reported to be preferable over printed, written information material (e.g., pamphlets), and may help increase patient satisfaction with the process of informed consent and other aspects of a clinical encounter.

Patient education is of paramount importance in dentistry and oral health care in general [12]. The nature of common dental diseases such as dental caries and periodontal disease, their strong behavioral and self-care components, and their chronic but preventable courses, make education and communication a foundational component of promoting oral health. Fundamentally, Albano and colleagues [5] argue that if patients better understand their illness and treatment, they should be able to improve their health behaviors, self-care techniques, and this may even reduce treatment-related expenditures. The potential may be even greater when education is aimed at parents’ and pertains to their children’s disease and surgical treatment [13]. Recent examples in pediatric dentistry illustrate the benefits of visual communication aids as tools to improve parents’ and pediatric patients’ understanding of dental conditions and associated treatments [13,14]. In a recent study, both video and physical models were found to be effective in improving the oral hygiene of hearing-impaired children [15].

This communication presents the development, deployment, and two real-life applications of a web-based data visualization informatics pipeline, SculptorHD, that aims to improve visual communication in dentistry and oral health care of children. The central goal of our effort was the creation of a three-dimensional (3D), lifelike model of the pediatric dentition that can be dynamically annotated to illustrate dental conditions (e.g., “cavities” or missing teeth), and existing or proposed restorative treatments (e.g., fillings and crowns). It is envisioned that the 3D model can be useful in clinical, research, and dental education settings, for one-on-one education with families, to illustrate clinical findings and proposed treatment plans, and facilitate the informed consent process. Additionally, we anticipate that it can be adapted to serve as an efficient visual tool for summarizing results of epidemiologic surveys or other dental research studies that produce tooth surface-level information. In the following sections, we present the pipeline’s design and customization features, performance, public deployment, and two real-life applications.

## 2. Materials and Methods

### 2.1. Conceptualization of the Application

**Figure 1:**
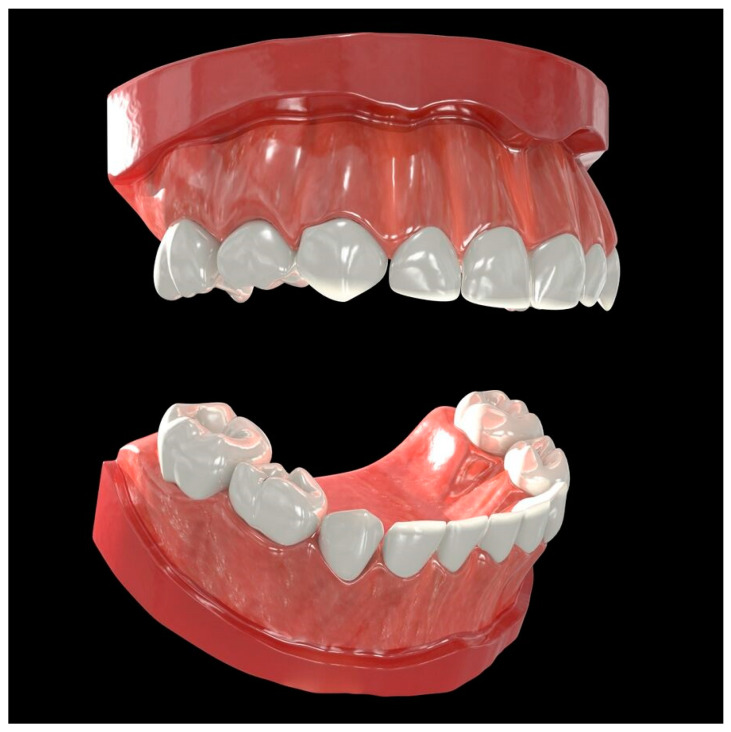
The original model file used in the prototype. A 3D model of the pediatric human dentition, composed of 72,597 total polygonal surfaces, and twenty-two unique textures.

The basic concept behind the development of SculptorHD is the conversion of tooth surface-level information generated by a clinical examination (i.e., an individual patient) or research studies (i.e., many study participants) to colorized, three-dimensional renderings. Our model includes the twenty teeth in the primary human dentition; these are segmented in 88 individual tooth surfaces, consistent with principles of dental anatomy, conventions of treatment planning and electronic patient records, and requirements of dental documentation. In the following paragraphs, we outline the methods for creating a pipeline which can quickly apply a subset of modifications to an existing 3D model (Figure 1). The modifications are defined in advance, are specific to the basic model, and may include, but are not limited to, color changes, regional opacity, and the way portions of the model reflect light. The last modification is used to represent different materials such as enamel (i.e., healthy tooth surface), metal, glass, porcelain, etc. Once modifications are defined, they can be applied automatically using a key that maps input data to modifications. The output is a derivative of the original model. The first step in building the modification interface is developing an understanding of the general structure of 3D models, and then more specifically the structure of the model to be modified. Because all modifications are based on this structure, we detail how each component controls or influences the model’s appearance.

### 2.2. Elements of the Baseline Model and Modifications

There are four main components which define the appearance of the 3D dentition model.

1. Mesh(s)—A geometric 3D structure made up of polygons,
2. Material(s)—Physical properties applied to whole meshes which influence the way their surface reflects or scatters light,
3. Texture(s)—One or more 2D images whose contents can be wrapped—in whole or in part—over specified regions of a mesh,
4. Light sources—responsible for illuminating the scene.

The core of our method involves the independent modification of these four properties. We posit that the design benefits greatly from a model whose parts are well organized and segmented prior to the implementation of various visual modifications. Specifically, the pediatric dentition model must be able to accommodate changes of each tooth material independently, to accommodate visualizing restorations such as stainless-steel crowns using a metal-like material placed on individual teeth. For this reason, it proved useful that each tooth could be a separate mesh, rather than an extrusion of the gingival mesh. If it were not already the case, the mesh could be cut along its polygons to allow for the changes. This feature enables each tooth to receive a unique material so that it can change independently of the other teeth in the dental arch. Each arch has its own mesh, but because there is no need for changing them independently, they are assigned the same soft tissue, gingiva-like material to reduce complexity and file size.

Perhaps the simplest modification of any 3D model is the adjustment of its surface color, i.e., textures. Almost all rendering engines default to a texture system known as UV mapping. This process is used to assign pixel locations on a 2D image to XYZ coordinates on a geometric mesh. The pixel location within the 2D image is denoted by U, V since X, Y, Z are already used on the mesh. If the intention is to change the model’s surface colors, the mapping system must be able to accommodate it. The process is complete when the entirety of the mesh is unfolded and laid flat across a known area of one or more 2D images. The images are linked to the model by filename, such that overwriting or changing “A.jpg” results in all surfaces linked to this texture to be redrawn accordingly.

**Figure 2:**
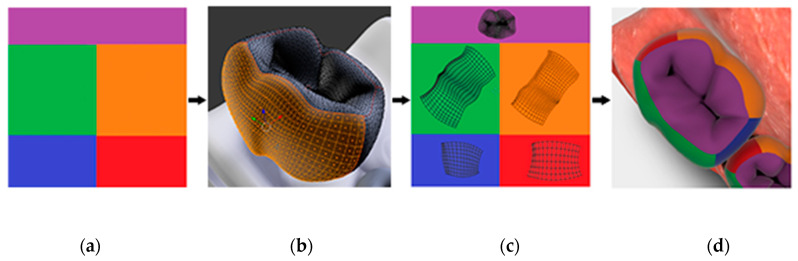
Representation of the UV mapping process for a single tooth: (**a**) Image file UV_T.PNG, pre-colored for convenience with desired subsections; (**b**) The mesh of tooth T being sectioned into surfaces of interest; (**c**) The mesh of tooth T cut and UV mapped within Tooth_T.PNG; (**d**) The rendered appearance of tooth T after completion of UV mapping.

The specifics of how many images are used and how the mesh is positioned within the image are heavily dependent on the model and the desired control scheme for changing its colors. The texture structure of the prototype pediatric dentition 3D model uses twenty image files, each named for one of the twenty primary teeth, such as ‘UV_A.png’. Within each of these image files we defined five rectangular subsections that represent features of dental anatomy, one for each individual tooth surface; five surfaces (i.e., occlusal, buccal, lingual, mesial, distal) for each posterior tooth (i.e., molar) and four surfaces (i.e., buccal, lingual, mesial, distal) for each anterior tooth (i.e., canines and incisors). This simplifies the process of UV mapping such that segmenting each tooth mesh along its line angles allows us to quickly place its flattened representation within a known subsection. This process is summarized in Figure 2.

Once the model structure is satisfactory and well understood, it can be exported. The prototype is exported using the GLB file format due to its ability to preserve this structure in a small file size.

### 2.3. Web Implementation of User Input Modifications

**Figure 3:**
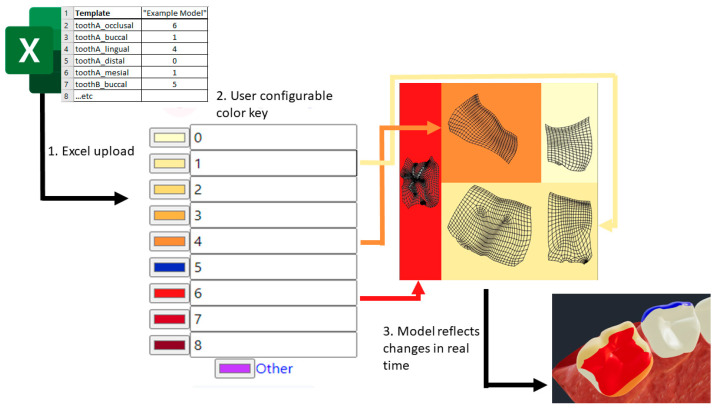
Process flow of data entry via a MS Excel template, its interpretation, and the customization of the process (i.e., color selection) via the web interface.

The remainder of the pipeline involves creating a web tool designed to represent user input into a defined set of modifications to the previously defined model components. The prototype uses the React JavaScript framework to define the user interface (UI) and transduction scheme. The following implementation steps are followed in sequence, as illustrated in Figure 3.

1. First, raw data are input into the system via an MS Excel file, which is translated into a JSON model object containing: an array of teeth, their corresponding surfaces, and the input values provided for these surfaces.
2. The system then uses a key to define how each input should appear when applied to the model. The key is updated in real-time in response to user input via the web UI and is used to transduce rawTooth JSON object into a new modeledTooth JSON object.
3. The modeledTooth object no longer contains the raw values and defines only properties which relate directly to the visual representation of a single mesh, in our example, a tooth. These properties include the tooth name, an encoded texture file, and the desired properties of the mesh’s associated material.
  All encoded texture files are provisioned by creating a new image via the HTML canvas element, coloring in the appropriate rectangular subsections seen in panel A of Figure 2 and assigning a reference to the texture file property of a modeledTooth.
4. In the last step, all modeledTooth objects are combined and passed to the rendering handler. The rendering handler is responsible for comparing an incoming modeledTooth to the one which currently exists in the rendered model, using the script three.JS to swap them wherever the two differ. The script three.JS handles displaying of the newly updated model and allows for packaging and retrieving a model file for download or upload to third party services.

### 2.4. Data Used for Clinical and Research Scenario Demonstrations

The clinical scenario used for demonstration is based on fictional data but closely resembles a sub-type of early childhood caries (ECC; a condition that is defined by the presence of dental caries lesions or “cavities” in a child under the age of 6) [16] wherein both anterior and posterior teeth are affected [17] and restorative treatment is indicated.

The data used for the research application and demonstration of SculptorHD were collected in the ZOE 2.0 pediatric oral health cohort, an IRB-approved (UNC-Chapel Hill IRB approval #14-1992) epidemiologic study of early childhood oral health in North Carolina (NC), United States [18,19]. The goal of ZOE 2.0 is to advance our understanding ECC and its multi-level determinants. Between 2016 and 2019, the study investigators collected a vast array of clinical information and biospecimens from a community-based sample of approximately 6500 preschool-age children ages 3–5, attending NC public preschools. A detailed description of the clinical data collection procedures and protocols has been previously reported by Ginnis et al. [20] and early analysis reports have been recently reported by Simancas-Pallares et al. [21] and Karhade et al. [22]. The motivation for the application of SculptorHD presented in this paper is that ECC manifests with distinct clinical presentations [17] that may affect different groups of teeth (e.g., anterior versus posterior, and upper versus lower) and may have different risk factors or etiologies. Traditional measures of caries experience, including the dmfs index (i.e., enumerating the number of caries-affected primary tooth surfaces), do not include any information besides the number of affected tooth surfaces. For the purposes of communicating distinct clinical patterns of ECC in a clinically interpretable manner, we used SculptorHD to summarize and illustrate the intra-oral presentation of caries experience among children with different clinical subtypes of ECC.

## 3. Results

The prototype SculptorHD tool has been deployed via the Google Cloud Platform App Engine and can be accessed via: https://sculptor.childrensoralhealth.org/ (accessed on 28 July 2021). In its current implementation, the tool can accommodate tooth surface-level input data in integer and proportion formats, using manual or MS Excel sheet data entry (a data input template is provided online). Outputs can be customized for clinical presentation (e.g., for patient education or treatment planning) or summarization of tooth surface conditions (e.g., research findings). The produced models are directly visualized on the web-app interface, can be downloaded as a gl transmission format (glTF) file, or be exported to third-party applications for further processing.

**Figure 4:**
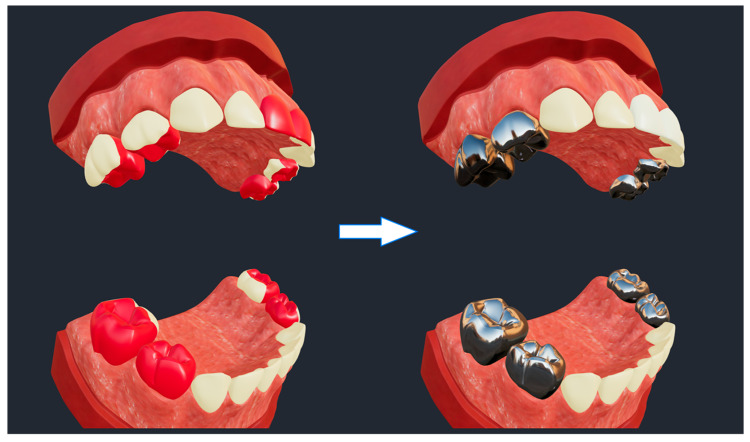
Representation of a simulated dental restorative treatment for a severe case of early childhood caries (i.e., multiple caries-affected primary teeth) that can be used for demonstration and educational purposes with families of patients. Caries lesions are displayed with red color in the model on the left-hand side (i.e., pre-treatment). The model on the right-hand side illustrates the restorative result that would be achieved using stainless-steel crowns for the restoration of posterior teeth and tooth-colored ceramic crowns for the restoration of the maxillary anterior teeth.

A lifelike clinical use scenario of SculptorHD is presented in Figure 4. The scenario is representative of a simulated dental restorative treatment for a severe case of early childhood caries (i.e., multiple caries-affected primary teeth in a child under the age of 6 years) [18] that can be used for demonstration and educational purposes with families of patients. In this case, tooth surfaces with caries lesions are displayed with red color in the model on the left hand-side (i.e., pre-treatment). The model on the right hand-side illustrates the restorative result that would be achieved using stainless-steel crowns for the restoration of posterior teeth (i.e., the eight primary molars, or teeth #55, #54, #64, #65, #75, #74, #84, and #85 according to the FDI nomenclature system) and tooth-colored ceramic crowns for the restoration of the maxillary anterior teeth (i.e., the four primary maxillary incisors, or teeth #52, #51, #61, and #62).

**Figure 5:**
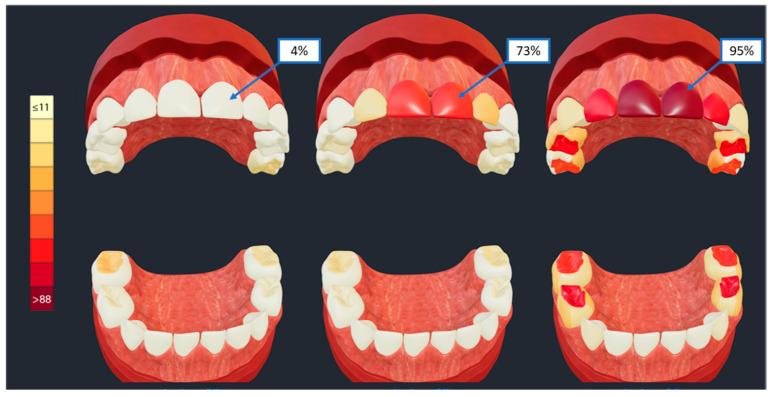
A research application of SculptorHD. Clinical subtypes of early childhood caries were generated using LCA of tooth-surface level caries experience data from over 6000 child participants of the ZOE 2.0 study. Tooth surface-level probabilities of caries experience within each disease subtype are displayed in these three representative models (i.e., maxillary central incisors have caries experience in 4% of children represented by the left model, 73% in the middle model, and 95% in the right model), enabling clinical interpretation: the models enable the illustration of caries affection of different sets of teeth and tooth surfaces and at varying levels of severity or frequency.

Second, we present a real-life research application of SculptorHD. Clinical subtypes of early childhood caries were generated using latent class analysis (LCA) of tooth-surface level caries experience data from over 6000 child participants of the ZOE 2.0 pediatric oral health cohort study [17]. Tooth surface-level probabilities of caries experience [18] within each disease subtype are displayed in these three representative models, enabling clinical interpretation. Specifically, the models enable the illustration of caries affection of different sets of teeth and tooth surfaces and at varying levels of severity or frequency, with a nine-level gradient red color (Figure 5, vertical color scale on the left) representing increments of 11% in the prevalence of caries experience on each individual surface. The three subtypes of ECC presented in Figure 5, are thus differentiated from each other by the prevalence of caries experience on the upper anterior teeth (i.e., maxillary central incisors) that ranges between 4% (left model) and 95% (right model); the right model also illustrates high prevalence of caries lesions on molar teeth compared to the other two models.

The prototype’s speed was then tested by importing and generating models from individual tooth surface-level data of 54 ECC subtypes (similar to those presented in Figure 5). The pipeline generated and subsequently displayed the corresponding 54 annotated 3D renditions of the primary dentition at an average rate of 956 ms per model with a standard deviation of 58 ms. This time is insignificant compared to the alternative approach of manually editing the base model—requiring not only specialized knowledge of the base model, but also orders of magnitude more time. Based these two real-life applications, we posit that the methods employed in SculptorHD and its web-based deployment are well-suited for the generation of lifelike 3D models of the pediatric dentition that can be used for numerous downstream applications, including clinical, patient education, and research, while allowing the further customization and post-processing of the produced models.

## 4. Discussion

Models have a history of helping us to better understand the world and in this paper, we introduce a customizable approach for producing better 3D models, with an immediate application in the oral health domain and specifically pediatric dentistry. We strongly believe that the model produced by SculptorHD will be practically useful as a communication aid for visualizing tooth-surface level conditions of the pediatric dentition in clinical, educational, and research settings. Besides the two real-life applications that we presented, at this time there are no empirical data to determine its utility, acceptability, or superiority over more established methods such as printed material [23]. In addition to its postulated attractiveness in presenting clinical findings and research results, we find that the tool is sufficiently fast and flexible to be deployed as a chairside communication aid and interfacing to an electronic patient record. It is also likely to become part of educational modules [24].

We must stress that the tool is not intended to replace direct, custom, intraoral 3D scans of individual patients. Obviously, the latter would be superior in terms of illustrating dental conditions with higher specificity and being entirely personalized. However, 3D scanning for documentation purposes alone is not yet routinely done in clinical and public health or research settings, and it may be particularly challenging among very young children. Nevertheless, we believe that our model might facilitate patient-provider interactions, and provider-provider interactions, through its ability to be readily accessible to all involved parties and its reliance on conventionally obtained, tooth surface-level data on dental caries, actual or planned restorations. Moreover, the tool is flexible enough to accommodate the illustration of a variety of conditions including non-carious tooth surface defects (i.e., hypoplastic defects may simply be presented with a different user-defined color or texture), findings from radiographic examinations (i.e., proximal caries lesions), as well as other potential pathologies (e.g., conditions of the pulp).

We have demonstrated in two applications the real-world value of such an approach and strongly believe that it has significant potential to improve overall quality dental care delivery. In conclusion, we have successfully designed, developed, and web-deployed a 3D visualization tool for the purpose of illustrating pediatric tooth surface conditions in life-like models of the pediatric dentition, including customizable user inputs and outputs. The tool has immediate applications and may enhance reporting of research studies and communication in clinical setting, although empirical data supporting this will need to be generated by future studies.

## Data Availability

De-identified clinical, questionnaire, and demographic data obtained in ZOE 2.0 are publicly available at: https://doi.org/10.17615/8yjy-w790 (accessed on 10 June 2021).
